# 44. Impact of Nursing Home (NH) Universal Decolonization and COVID Prevention Training on COVID-19 Burden During the 2020-2021 Winter Surge in Orange County (OC), California (CA)

**DOI:** 10.1093/ofid/ofab466.044

**Published:** 2021-12-04

**Authors:** Gabrielle Gussin, Raveena Singh, Shruti K Gohil, Raheeb Saavedra, Thomas Tjoa, Robert Pedroza, Chase Berman, Jessica Park, Kimia Ghasemian, Avy Osalvo, Joshua Hsi, Emily A Hsi, Stephanie Chun, Matthew Zahn, Emily Fonda, Susan S Huang

**Affiliations:** 1 University of California, Irvine; 2 Univeristy of California, Irvine, Irvine, California; 3 UC Irvine School of Medicine, IRVINE, California; 4 Orange County Department of Health, Irvine, California; 5 CalOptima, Orange, California

## Abstract

**Background:**

OC is the 6^th^ largest U.S. county with 70 NHs. Universal decolonization (chlorhexidine for routine bathing, and twice daily nasal iodophor Mon-Fri every other week) was adopted in 24 NHs prior to the COVID-19 pandemic, and 12 NHs (11 of those adopting decolonization) participated in a COVID prevention training program with a rolling launch from July-Sept 2020. We evaluated the impact of these initiatives on staff and resident COVID cases.

**Methods:**

We conducted a quasi-experimental study of the impact of decolonization and COVID prevention training on staff and resident COVID cases during the CA winter surge (11/16/20-1/31/21), when compared to non-participating NHs. Decolonization NHs received weekly visits for encouraging adherence during the pandemic, and NHs in the COVID training program received 3 in-person training sessions for all work shifts plus weekly feedback about adherence to hand hygiene, masking, and breakroom safety using video monitoring. We calculated incident 1) staff COVID cases, 2) resident COVID cases, and 3) resident COVID deaths adjusting for NH average daily census. We assessed impact of initiatives on these outcomes using linear mixed effects models testing the interaction between any training participation and calendar date when clustering by NH. Because of the overlap of the two initiatives, we evaluated ‘any training’ vs ‘no training.’

**Results:**

63 NHs had available data. 24 adopted universal decolonization, 12 received COVID training (11 of which participated in decolonization), and 38 were not enrolled in either. During the winter surge, the 63 NHs experienced 1867 staff COVID cases, 2186 resident COVID cases, and 251 resident deaths due to COVID, corresponding to 29.6, 34.7, and 4.0 events per NH, respectively. In NHs participating in either initiative, staff COVID cases were reduced by 31% (OR=0.69 (0.52, 0.92), P=0.01), resident COVID cases were reduced by 43% (OR=0.57 (0.39, 0.82), P=0.003), and resident deaths were reduced (non-significantly) by 26% (OR=0.74 (0.46, 1.21), P=0.23).

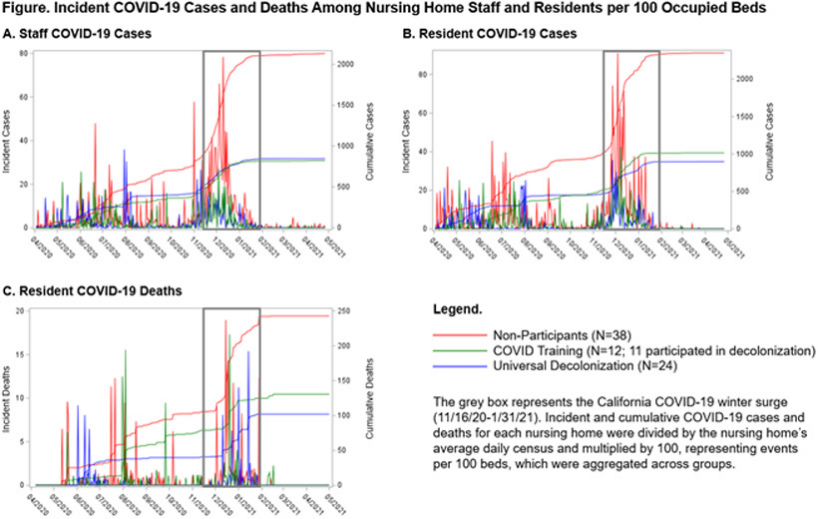

The grey box represents the California COVID-19 winter surge (11/16/20-1/31/21). Incident and cumulative COVID-19 cases and deaths for each nursing home were divided by the nursing home’s average daily census and multiplied by 100, representing events per 100 beds, which were aggregated across groups.

**Conclusion:**

NHs are vulnerable to COVID-19 outbreaks. A universal decolonization and COVID prevention training initiative in OC, CA significantly reduced staff and resident COVID cases in this high-risk care setting.

**Disclosures:**

**Gabrielle Gussin, MS**, **Medline** (Other Financial or Material Support, Conducted studies in which participating hospitals and nursing homes received contributed antiseptic and cleaning products)**Stryker (Sage**) (Other Financial or Material Support, Conducted studies in which participating hospitals and nursing homes received contributed antiseptic products)**Xttrium** (Other Financial or Material Support, Conducted studies in which participating hospitals and nursing homes received contributed antiseptic products) **Raveena Singh, MA**, **Medline** (Other Financial or Material Support, Conducted studies in which participating hospitals and nursing homes received contributed antiseptic and cleaning products)**Stryker (Sage**) (Other Financial or Material Support, Conducted studies in which participating hospitals and nursing homes received contributed antiseptic products)**Xttrium** (Other Financial or Material Support, Conducted studies in which participating hospitals and nursing homes received contributed antiseptic products) **Shruti K. Gohil, MD, MPH**, **Medline** (Other Financial or Material Support, Co-Investigator in studies in which participating hospitals and nursing homes received contributed antiseptic and cleaning products)**Molnycke** (Other Financial or Material Support, Co-Investigator in studies in which participating hospitals and nursing homes received contributed antiseptic and cleaning products)**Stryker (Sage**) (Other Financial or Material Support, Co-Investigator in studies in which participating hospitals and nursing homes received contributed antiseptic and cleaning products) **Raheeb Saavedra, AS**, **Medline** (Other Financial or Material Support, Conducted studies in which participating hospitals and nursing homes received contributed antiseptic and cleaning products)**Stryker (Sage**) (Other Financial or Material Support, Conducted studies in which participating hospitals and nursing homes received contributed antiseptic products)**Xttrium** (Other Financial or Material Support, Conducted studies in which participating hospitals and nursing homes received contributed antiseptic products) **Robert Pedroza, BS**, **Medline** (Other Financial or Material Support, Conducted studies in which participating hospitals and nursing homes received contributed antiseptic and cleaning products) **Chase Berman, BS**, **Medline** (Other Financial or Material Support, Conducted studies in which participating hospitals and nursing homes received contributed antiseptic and cleaning products) **Susan S. Huang, MD, MPH**, **Medline** (Other Financial or Material Support, Conducted studies in which participating hospitals and nursing homes received contributed antiseptic and cleaning products)**Molnlycke** (Other Financial or Material Support, Conducted studies in which participating hospitals and nursing homes received contributed antiseptic and cleaning products)**Stryker (Sage**) (Other Financial or Material Support, Conducted studies in which participating hospitals and nursing homes received contributed antiseptic and cleaning products)**Xttrium** (Other Financial or Material Support, Conducted studies in which participating hospitals and nursing homes received contributed antiseptic and cleaning products)

